# Integrative Transcriptomic and Target Metabolite Analysis as a New Tool for Designing Metabolic Engineering in Yeast

**DOI:** 10.3390/biom14121536

**Published:** 2024-11-30

**Authors:** Alejandro Lopez-Barbera, Nerea Abasolo, Helena Torrell, Nuria Canela, Salvador Fernández-Arroyo

**Affiliations:** Centre for Omic Sciences, Eurecat, Centre Tecnològic de Catalunya, Joint Unit Eurecat—Universitat Rovira i Virgili, Unique Scientific and Technical Infrastructure (ICTS), 43204 Reus, Spain; alejandro.lopez@eurecat.org (A.L.-B.); nuria.canela@eurecat.org (N.C.)

**Keywords:** CRISPR/Cas9, isoprenoids, metabolic engineering, natural products, targeted metabolite analysis, mevalonate, *Saccharomyces cerevisiae*, synthetic biology, transcriptomics

## Abstract

Precision fermentation processes, especially when using edited microorganisms, demand accuracy in the bioengineering process to maximize the desired outcome and to avoid adverse effects. The selection of target sites to edit using CRISPR/Cas9 can be complex, resulting in non-controlled consequences. Therefore, the use of multi-omics strategies can help in the design, selection and efficiency of genetic editing. In this study, we present a multi-omics approach based on targeted metabolite analysis and transcriptomics for the designing of CRISPR/Cas9 in baker’s yeast as a more efficient strategy to select editing regions. Multi-omics shows potential to reveal new metabolic bottlenecks and to elucidate new metabolic fluxes, which could be a key factor in minimizing the metabolic burden in edited microorganisms. In our model, we focus our attention on the isoprenoid synthesis due to their industrial interest. Targeted metabolite detection combined with a transcriptomic analysis revealed hydroxymethylglutaryl-CoA reductases (HMGs) as the best target gene to induce an increase in isoprenoid synthesis. Thus, an extra copy of *HMG1* was introduced using, for the first time, the synthetic *UADH1* promoter. The multi-omics analysis of the recombinant strain results in an accurate assessment of yeast behavior during the most important growth phases, highlighting the metabolic burden, Crabtree effect or the diauxic shift during culture.

## 1. Introduction

Precision fermentations involve the rewiring of metabolic pathways in safe microorganisms to produce food ingredients, cosmetical compounds or any kind of metabolites with industrial relevance from abundant and inexpensive substrates [1]. This rewiring of metabolic pathways is produced by engineering native pathways by downregulating or upregulating genes already present in the genome or by introducing a non-native pathway from other organisms into a more manageable and cheaper maintenance organism. These modifications in the genome of the organisms that involve the manipulation of enzymatic, transport and regulatory functions, by using recombinant DNA, are known as metabolic engineering [2,3]. Clustered Interspaced Short Palindromic Repeats (CRISPR)-based methods have become a popular recombinant DNA technique with academic, industrial and therapeutic potential [4], especially when using the RNA-guided DNA endonuclease enzyme CRISPR-associated protein 9 (Cas9). One main advantage of CRISPR against other types of gene-editing systems is the specificity to target a determined sequence rather than on random integration. The basic principles of CRISPR design involve identifying a suitable target region or regions [5]. This can be challenging, as the metabolic fluxes emerge from the complex interactions between pathways, metabolites feedback regulations, enzymes regulation and environmental changes [6]. In this complex biological network, the selection of the most suitable target region to apply CRISPR is tricky. Additionally, even when the metabolite production has been improved, it still creates a highly metabolic burden due to the increased biosynthesis demand that reduces the potential production of the engineered cell [7].

In this study, we propose an omics analysis based on transcriptomic and targeted metabolite analysis as a potential tool to select the specific genes to improve the metabolic pathways towards desired metabolites. Firstly, by comparing the target metabolite(s) of the microorganism inoculated in different supplemented media designed to enhance a specific pathway, we expect that an increase in the metabolic activity will lead to a higher production and accumulation of the final product or intermediates. Secondly, a transcriptomic analysis comparing the gene expression of the microorganism grown in two completely different media (with low and high nutrient levels, respectively, thus affecting the metabolic activity rate of the microorganism) will help us to elucidate the different expression patterns involved in the target pathway [8], identifying the potential candidate genes for CRISPR/Cas9 editing. After metabolic engineering, analyzing the metabolome of the edited cells can provide us information about the new phenotype induced by the editing. This information can be used to re-adjust the media to meet the new cell requirements. Finally, a transcriptomic analysis post-editing can provide information about the changes in the expression levels and how the cell is dealing with the alterations in the biosynthesis activity. These tools are useful for validating the editing, characterizing the model, revealing further mechanisms to enhance even more the desired production and preventing the excessive metabolic burden.

In this context, transcriptome profiling has demonstrated its capacity to identify differential gene expression in response to environmental changes or different stages of cell development [9]. It has also been used to identify key genes and pathways corresponding to different stress conditions, environmental responses and cell cycles [10,11]. Additionally, transcriptomics has been used to analyze differences in mRNA of CRISPR/Cas9-mutated *Saccharomyces cerevisiae* (*S. cerevisiae*), showing that three knockdown genes using CRISPR/Cas9 lead to a different expression of up to 570 genes [12]. However, the mRNA levels by themselves do not correlate to the protein activity and, as a result, neither with the phenotype. For this reason, the addition of targeted or untargeted metabolomics studies that provide the closest representation of the phenotype [13] can add valuable information based on changes in the environment and/or the genome [14,15]. In different studies, metabolic profiling has been used to discriminate between different types of Mezcal and wines produced in different regions, with different ingredients and different processes, as well as the strains of yeast used to produce wine [16,17]. In addition, an integration of a transcriptomic–metabolomic analysis has shown the potential to reveal key metabolic pathway responses under environmental stress [18].

**Figure 1:**
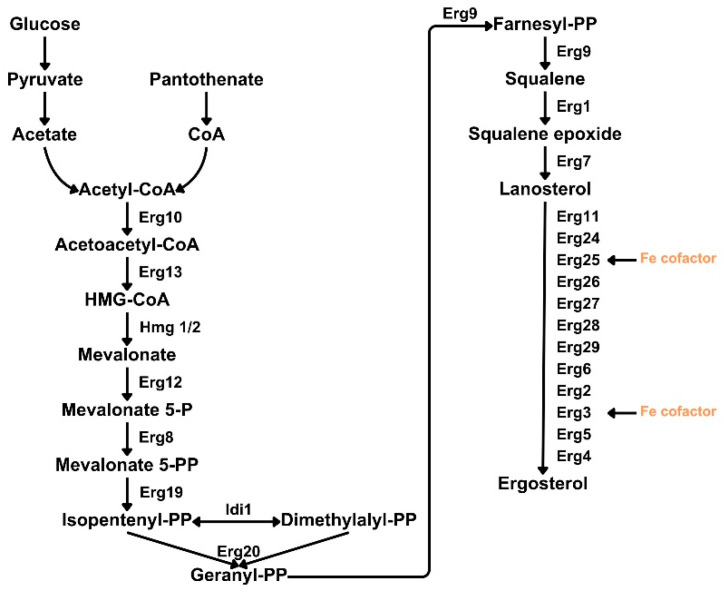
Overview of the ergosterol pathway. Fe cofactor represents enzymes that are dependent on iron. Metabolite abbreviations: Coenzyme A (CoA), 3-hydroxy-3-methylglutaryl coenzyme A (HMG-CoA), phosphate (P) and pyrophosphate (PP).

The combination of a multi-omics approach with CRISPR has been theorized to be a promising tool to provide a wide-range dataset that can serve as a blueprint for checking target and off-target effects and fully characterize the modified organisms [19,20]. For this purpose, we selected a well-characterized microorganism, the baker’s yeast *Saccharomyces cerevisiae*, that has been widely manipulated for its use in the bioproduction of different chemicals [21,22]. One of the most studied pathways in *S. cerevisiae*, with a wide use in the pharmaceutical sector, is the mevalonic acid (MVA) pathway, not only for the importance of MVA itself as an intermediate in the production of statins, antibiotics, antifungals or anticancer agents, but also because it is the backbone for sterol biosynthesis (Figure 1). However, the production of MVA using wild-type strains is very inefficient for the industrial requirements, and different approaches to improve its efficiency have been developed [23].

Therefore, we consider incorporating the transcriptomics along a targeted metabolite analysis during a CRISPR/Cas9 design as it provides necessary tools and knowledge to improve and optimize biotechnological production. This approach could identify potential genes in which an editing could increase the production of mevalonate in *Saccharomyces cerevisiae* and, at the same time, allow one to fully characterize the changes in the model beyond the mevalonate (MVA) pathway.

## 2. Methods

### 2.1. Yeast Culture

*S. cerevisiae* strain S288C (*MATα*, *SUC2*, *gal2*, *mal2*, *mel*, *flo1*, *flo8-1*, *hap1*, *ho*, *bio1* and *bio6*) was obtained from LCG standards (Teddington, UK) and used as starting strain and as a gene donor. This strain was cultured in different media to stimulate isoprenoid production and to screen for genes involved in the process. For this purpose, *S. cerevisiae* was inoculated in two different media, a starvation minimum medium (M1) composed with 0.67 g/L yeast nitrogen base (YNB), amino acids (Y1250 at final concentration of 10 mg/mL of L-histidine, 20 mg/L of DL-methionine and 20 mg/mL of DL-tryptophan) and a control medium (M2) using a yeast extract peptone dextrose (YPD) medium (10 g/L yeast extract, 20 g/L peptone and 20 g/L glucose).

Furthermore, to compare the MVA production in different conditions, 7 more media (M3 to M9) were designed (Supplementary Table S1), where *S. cerevisiae* culture was maintained for 72 h. Media supplementation was based on 4 compounds added to the YPD media: an extra amount of glucose as carbon source; iron (II) as cofactor for the enzymes involved in the isoprenoid synthesis [24]; pantothenate (Vitamin B5), a precursor of coenzyme A (CoA), to bypass the rate-limiting step in the CoA pathway that restricts the sterols biosynthesis [25]; and pyruvate as an extra carbon source and as the closest precursor of acetyl-CoA [26]. All reagents and media were obtained from Merck (Darmstadt, Germany). All cultures were incubated at 30 °C with shaking (220 rpm). Samples of 1.5 mL from each culture were collected at 2 h, 4 h, 6 h, 8 h, 12 h, 2 4 h, 48 h and 72 h, centrifuged for 5 min at 8000× *g*, and, after discarding the supernatant, pellets were stored at −80 °C until further processing.

### 2.2. Repair Template Construction

Yeast DNA was extracted from 5 mL YPD culture using a DNeasy PowerSoil Pro Kit (Qiagen Inc., Hilden, Germany) according to the manufacturer’s instructions and incubated overnight at 30 °C and 220 rpm. The primers (Supplementary Table S2) were synthesized by Condalab (Torrejon de Ardoz, Spain).

**Figure 2:**
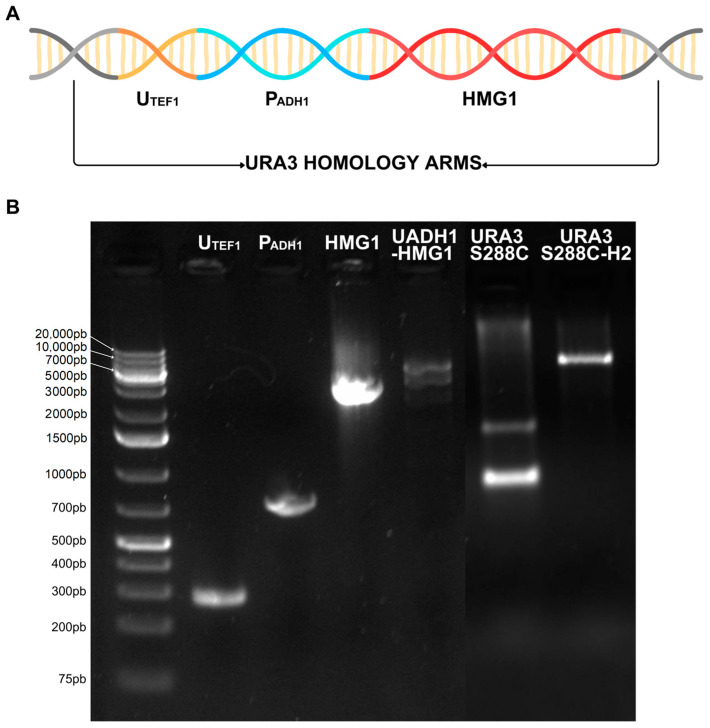
Construction of the recombinant strain by CRISPR. (**A**) Scheme of the composition of the repair template *UADH1-HMG1*. From left to right: 5′ *URA3* homology arm, *U_TEF1_*, *P_ADH1_*, *HMG1* and 3′ *URA3* homology arm. (**B**) Electrophoresis of the different components of the repair template and recombinant validation in 1% agarose gel and GeneRuler 1 Kb plus (Thermo Fisher Scientific, Waltham, MA, USA). In order: *U_TEF1_* (284 pb), *P_ADH1_* (756 pb), *HMG1* (3.2 Kb) and *UADH1-HMG1* (4.25 Kb). *URA3* gene amplification from S288C strain (800 pb) and same region amplification from the recombinant strain (4.92Kb). Original images of (**B**) can be found in supplementary materials (Supplementary Figure S1).

*HMG1* gene was fused under the regulation of a semiartificial strong housekeeping promoter, named *UADH1*. This promoter was described for the first time by Zhou, C. et al. [27], constructed using two different sequences, the upstream activation sequences of the native promoter of the translation elongation factor-1α (*TEF1*) *U_TEF1_*, and combined with the native promoter of the alcohol dehydrogenase I (*ADH1*) *P_ADH1_* (Figure 2A). This fused promoter has only been used previously to calculate *UADH1* activity by binding it to the green fluorescent protein gene and measuring the expression of the protein [27]. The final construct was named *UADH1-HMG1* and was 4.3 kb long flanked by 80 pb homologous region to *URA3* gene (orotidine 5′-phosphate decarboxylase) (Figure 2B). The repair template was constructed by overlapping PCR [28].

All the PCR and OE-PCR were performed using the KAPA HiFi HotStart obtained from Roche Diagnostics Corporation (Indianapolis, IN, USA).

### 2.3. Plasmid Ligation

Plasmid pML104-KanMx4, a modification of the pML104 described by Laughery et al. [29], that substituted the URA3 selection marker for KanMX, which confers *S. cerevisiae* resistance to antibiotic G418, was a gift from Patricia Wittkopp (Addgene plasmid #83476; http://addgene.org/83476, accessed on 29 November 2024; RRID: Addgene_83476). The plasmid carries the genes to produce Cas9 protein and a multiple cloning site to introduce the gRNA sequence (gDNA) under the expression of *SNR52* promoter. It also contains the AmpR gene for ampicillin resistance. Antibiotics for selection were obtained from Merck.

For gDNA integration in the plasmid, the Basic Protocol 1 from Laughery and Wyrick was used [30]. The gDNA containing the recognition sequence of restriction enzyme BclI into the 5′, the 20 bp for Cas9 recognition and the structural sequence of tracrRNA in the 3′ was synthesized by Condalab. The 20 bp for Cas9 recognition was designed to match the *URA3* gene of *S. cerevisiae* (Supplementary Figure S2).

After insertion, the plasmid was transformed by heat shock into *E. coli DH5α* Competent Cells from Thermo Fisher Scientific (Waltham, MA, USA) and plated in Luria-Bertani Lennox Agar with ampicillin 100 μg/mL. Plasmids were extracted from selected colonies using QIAprep^®^ Spin Miniprep Kit (Qiagen Inc.), according to the manufacturer’s instructions, and sequenced by Sanger to confirm the correct integration of the gDNA.

### 2.4. CRISPR/Cas9

The Cas9 gene was inserted into S288C cells using the plasmid pML104-KanMx. gDNA was designed to perform a double-strand break inside the native *URA3* gene making the recombinant cells auxotroph for uracil.

A co-transformation of the gDNA integrated pML104-KanMx4 and *UADH1-HMG1* into *S. cerevisiae* S288C was performed using the Lithium Acetate/Single-Stranded Carrier DNA/Polyethylene Glycol method [31] with an extra step of adding 1 mL of YPD and incubating during 3 h for KanMx expression before selection with G418. All reagents were obtained from Merck.

The selection of the transformed yeasts was performed by plating in agar YPD plaques with 300 μg/mL of G418. Transformed cells will be G418-resistant. The plates were incubated for 3 days at 30 °C.

CRISPR/Cas9 mutants were confirmed by PCR of the *URA3* gene and by growth in SC-Medium without Uracil. Genomic DNA from mutants was extracted using DNeasy PowerSoil Pro Kit (Qiagen Inc.) according to the manufacturer’s instructions. *URA3* gene was obtained by PCR from mutant and wild-type yeast genome, and gene length was compared using an electrophoresis in 1% agarose gel.

Mutant yeast genome was sequenced using MinION Flow Cell R9.4.1 from Oxford Nanopore Technologies (Oxford, UK) to confirm the correct incorporation of the repair template in the genome. DNA repair, end-prep, barcode ligation, adapter ligation and clean-up steps were performed following the SQK-LSK109 protocol (Oxford Nanopore Technologies). The final product was quantified using a Qubit fluorometer (Thermo Fisher Scientific) and loaded into the flow cell, with sequencing performed according to the manufacturer’s instructions.

### 2.5. Mevalonate Quantification Through Mass Spectrometry

Cell pellets were resuspended with 500 μL of water:methanol (1:2) with 2.65 μM of mevalonic acid (methyl-D3) (Merck) as internal standard. Afterwards, samples were frozen in liquid nitrogen for 1 min and thawed for 1 min and then sonicated for 2 min. This process was repeated 3 times, followed by vortex and centrifugation at 15,000× *g* for 10 min at 4 °C. The supernatant was transferred into 2 mL centrifuge tubes and dried in a Savant SPD2010 SpeedVac rotatory vacuum system (Thermo Fisher Scientific) and, finally, reconstituted in 100 μL of 10 nM ammonium acetate and placed into chromatographic vials.

For the calibration curve, mevalonic acid (Merck) was dissolved in 10 nM ammonium acetate to obtain 7 seriated concentrations (range from 0 to 165 μM) containing 2.65 μM internal standard and placed into glass vials for analysis. Calibration curves were plotted using the relative response (ordinate axis) and relative concentration (abscise axis) to the internal standard.

Samples and calibration curve (5 μL) were injected into a 1290 Infinity ultra-high-pressure liquid chromatograph (UHPLC) coupled with a dual Agilent JetStream electrospray ionization (ESI) source to a 6490 triple quadrupole mass spectrometer (QQQ-MS) (Agilent Technologies, Santa Clara, CA, USA). The UHPLC system was equipped with a binary pump (G7120A), an autosampler (G1316C) termostatized at 4 °C, and a InfinityLab Poroshell 120 HILIC-Z 2.7 μm, 2.1 mm × 100 mm column (Agilent Technologies). The mobile phase consisted of A: water with 10 nM ammonium acetate and 5 μM InfinityLab Deactivator Additive (Agilent Technologies); B: acetonitrile–water (9:1) with 10 nM ammonium acetate and 5 μM InfinityLab Deactivator Additive, at a flow rate of 0.25 mL/min. The gradient used was as follows: 0 min, 100% B; 2 min, 90% B; 4 min, 75% B; 10 min, 70% B; 15 min, 60% B; 18 min, 60% B; 18.5 min, 100% B. A post-run of 6.5 min in initial conditions was used for column conditioning. ESI source parameters were set as follows: gas temperature at 200 °C; gas flow at 11 L/min; nebulizer at 35 psi; sheath gas heater at 375 °C; sheath gas flow at 11 L/min; capillary (−) at 3500 V. QqQ-MS, working in negative mode, was used to detect and quantify MVA using the multiple reaction monitoring (MRM) mode: MVA parental ion at *m*/*z* 147 and product ions at *m*/*z* 59 and 57 (using collision energy at 8 and 16 V, respectively; MVA-(methyl-D3) parental ion at *m*/*z* 150 and product ions at *m*/*z* 59 and 60 (using collision energy at 8 and 12, respectively).

For the statistical analysis of metabolite results, non-parametric Mann–Whitney and Kruskal–Wallis tests (*p*-value < 0.05) were performed in Prism 9.5 (GraphPad, Boston, MA, USA).

### 2.6. Transcriptomic Analysis

The RNA was extracted from the yeast grown in M1 and M2 media samples. The lysis was performed using a combination of sonication at 20 kHZ with a 50% amplitude for 10 s + TRIzol^®^ Reagent (Invitrogen, Waltham, MA, USA) following manufacturer’s instructions. RNA purification was performed using PureLink RNA Mini Kit™ (Invitrogen) following manufacturer’s instructions.

RNA concentration and purity was determined using the NanoDrop™ 2000 spectophotometer (Thermo Fisher Scientific). RNA integrity was determined by capillary electrophoresis at the TapeStation using Agilent High Sensitivity RNA ScreenTape Assay (Agilent).

Expression of *HMG1* was quantified by quantitative PCR (qPCR) using ACT1 as reference gene [32]. cDNA was obtained by reverse-transcription PCR (RT-PCR) using SuperScript™ IV from Invitrogen (Thermo Fisher Scientific) following manufacturer’s instructions. TaqMan^®^ Gene Expression Assays for *HMG1* and *ACT1* as well as TaqMan Fast Advanced Master Mix were obtained from Thermo Fisher Scientific. qPCR was performed following manufacturer’s instructions.

Illumina Stranded mRNA libraries were prepared using the TruSeq Stranded mRNA kit (Illumina, San Diego, CA, USA) according to the manufacturer’s instructions and sequenced at pair-end 76 bp using the Illumina NextSeq2000 instrument. All samples surpass the threshold of 25M pair-end reads required for accurate and quality results. Raw fastq.gz files are publicly available in the ArrayExpress collection of BioStudies database (http://www.ebi.ac.uk/biostudies/arrayexpress, accessed 29 November 2024) under accession number E-MTAB-14650. Total high-quality reads were aligned against the GCF_000146045.2 *Saccharomyces cerevisiae* reference genome using HISAT2 version 2.2.1 (Lyda Hill Department of Bioinformatics, University of Texas Southwestern Medical Center, Dallas, TX, USA, and Center for Computational Biology, Johns Hopkins University, Baltimore, MD, USA) and annotated using StringTie 2.2.1 (Center for Computational Biology, Johns Hopkins University, Baltimore, MD, USA). The resulting estimated gene counts (Supplementary Tables S3 and S4) were further analyzed using DESeq2 after Trimmed Mean of M-values normalization in the ExpressAnalyst online platform [33]. Gene Set Enrichment Analysis (GSEA) was performed using the previously mentioned gene counts and using KEGG for pathway database in the GSEA software [34,35].

## 3. Results and Discussion

### 3.1. Transcriptomic Analysis of MVA Pathway in S. cerevisiae Wild-Type S288C

Acetyl-CoA is the direct precursor of the MVA pathway. Based on this, we decided to compare the gene expression levels of *S. cerevisiae* S288C in a nutrient-stress medium based on glucose depletion (M1) against a non-restrained medium (M2). The transcriptomic comparative of M1 and M2 showed differences in the expression of 1278 genes (*p*-value < 0.05, FDR) of which 667 were downregulated and 611 were upregulated in the minimum medium (M1) compared to the control medium (M2). Most downregulated genes were principally those involved in ribosome biogenesis, confirming that the cells are under stress conditions and kept in the stationary phase [36]. M1 stress was designed to be induced by carbon starvation. This was correlated with the transcriptome analysis using the hexose transport (HXT) genes, which are differently regulated according to the concentration of glucose in the media [37]. Thus, HXT1 and HXT3 genes, which are induced by glucose concentration, were downregulated, and, conversely, we observed an upregulation of HXT4, which is induced by low glucose concentrations [37]. We also observed an upregulation of the glucose-responsive transcription factor RGT1, which regulates the expression of the HXT genes. This RGT1 factor, only in absence of glucose, recruits the co-repressors Ssn6-Tup1 and establishes a direct repression over the mediator complex of RNA polymerase II [38], inducing the mentioned downregulation in the ribosome biogenesis [39]. We also observed a downregulation of HXK2 (hexokinase isoenzyme), which is the main hexokinase that catalyzes the phosphorylation of glucose in the cytosol, during cell growth on a glucose-rich medium [40]. Moreover, HXK1 and GLK1 (glucokinase), which also catalyze the phosphorylation of glucose, are upregulated, indicating glucose depletion [41]. These stress conditions reduce the growth capacity of S288C and, since ergosterol synthesis is increased during exponential growth [42], we expected to see differences in transcriptome of the sterol biogenesis, specifically in the MVA pathway, as our objective is to increase the mevalonate production.

**Figure 3:**
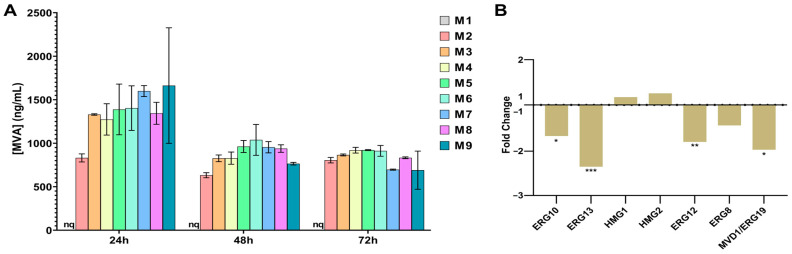
Wild-type S288C. (**A**) Differences in the transcript levels of the seven enzymes involved in the first six steps of the MVA pathway in the M1 media compared to the M2 media at 24 h. (**B**) MVA quantification in 9 different media during 24 h, 48 h and 72 h. * *p*-value < 0.05; ** *p*-value < 0.005; *** *p*-value < 0.001.

As expected, the ergosterol pathway was significantly downregulated. When we focused our attention on the genes that codify for enzymes in the MVA pathways, we observed a general downregulation under the M1 conditions, except for three enzymes without significant changes: the phosphomevalonate kinase ERG8 and 3-hydroxy-3-methylglutaryl coenzyme A (HMG-CoA) reductases 1 and 2 (Figure 3B). ERG8 transforms MVA-5-phosphate into MVA-5-pyrophosphate. This enzyme was slightly under-expressed but not statistically significant. However, since this enzyme is after mevalonate synthesis, we did not consider it as a good candidate for MVA overexpression by CRISPR. The second and third exceptions were the HMG-CoA reductases HMG1 and HMG2, which do not seem to be transcriptionally regulated under nutrient stress. It has been previously described that HMG1 and HMG2 in yeast are actually not regulated at the transcriptional level but at the translational and protein level [43]. This is in line with our results because, although mevalonate production in M1 was dramatically lower than in M2, the transcriptional level of the enzymes remained unchanged. Both enzymes convert HMG-CoA into MVA. Since our objective was to increase MVA production, we selected these enzymes as good candidates to be overexpressed by CRISPR. This is also consistent with the literature as the conversion of HMG-CoA to MVA, catalyzed by the enzyme HMG-CoA reductase, has been identified as the key rate-limiting step of the route. It is not surprising, then, that the overexpression of HMG1 and HMG2 has been a general and recurrent strategy for enhancing isoprenoid production [44,45,46].

### 3.2. Analysis of MVA Production in S. cerevisiae Wild-Type S288C

MVA pathway is the first step in the production of ergosterol, which is essential for the building and maintenance of the cell membrane, the maintenance of mitochondrial DNA, and the proliferation of yeasts. Based on previous transcriptomic results, we focused our attention on the MVA levels as a key step on isoprenoid production. In aerobic conditions, *S. cerevisiae* tends to produce isoprenoids despite the highly energy-consuming process. Conversely, during anaerobic conditions, hyperosmotic stress or iron deficiency, the production of isoprenoids decreases [47]. Thus, isoprenoid levels are dependent on the composition of the growth media. Based on this information, and to assess MVA production under different conditions, we grew *S. cerevisiae* in nine culture media differing in their nutritional supplementation. As expected, MVA concentration in M1 (minimum medium) was detectable at 24 h, 48 h and 72 h but under the limit of quantification. However, MVA was quantifiable in the rest of the enriched media, being the M9 medium (with the higher supplementation including Fe^2+^, glucose, pantothenate and pyruvate), the one with the highest concentration of MVA (Figure 3A). After 24 h, the concentration of MVA decreased and then stabilized, probably because the late stationary phase was reached, as free sterol intermediate production is lower during this phase [48].

Regardless of the increment of MVA accumulation in our model (producing 15 mg/L in 24 h), this concentration of MVA is far from other strains, e.g., the constructed by Wegner et al., 2021 [49], achieving 7.5 g/L after 48 h in a lab-scale bioreactor by using the exogenous mevalonate cassette of *Enterococcus faecalis* combined with the overexpression of CAB1 and the repression of ERG9. Although the concentration of MVA did not achieve these values, our objective to show the potential of transcriptomic and target metabolite analysis not only to fully characterize the post-edited strand but also during CRISPR/Cas9 design remains valid.

### 3.3. CRISPR-Cas9 Editing

After considering the transcriptomic and metabolic analysis, we decided to overexpress the HMGR enzymes in *S. cerevisiae*, specifically HMG1, which accounts for 90% of HMG-CoA reductase activity under aerobic conditions [50]. Thus, we introduced another copy of HMG1 under the regulation of a modified housekeeping promoter, *ADH1*, incorporating, for the first time, the upstream region of *TEF1* promoter (*UADH1*). *UADH1* has been previously used to prove the downregulation effect of the *marO* motif in *Escherichia coli* and validated in *S. cerevisiae* through the expression of the green fluorescent protein [27] but has never been used to overexpress engineered enzymes. Then, we decided to use this fused promoter, for the first time, for the expression of a new *HMG1* copy using the synthetic *UADH1* promoter, expecting to produce higher concentrations of mevalonate.

Despite the short homology arms of only around 50 pb for a 4.3 Kb long fragment, we successfully obtain one recombinant colony. PCR of the *URA3* gene of this colony showed a product size of approximately 5 kb as revealed by electrophoresis, which is similar to the repair template (4.3 Kb) and *URA3* gene (0.7 Kb in the wild-type strain) (Figure 2B). Auxotroph recombinant colonies for uracil were selected during a growth assay by replicas performed in the SC medium with and without uracil supplementation.

To confirm the DNA of the recombinant strain, the *URA3* gene was sequenced using Next-Generation Sequencing, revealing that the *URA3* gene was interrupted by the *UADH1-HMG1* sequence. Moreover, to confirm the duplication of *HMG1* gene, a qPCR was performed in M2 after overnight culture. As expected, *HMG1* expression showed a 6.41-fold increase in the mutant compared to the S288C wild-type (Supplementary Figure S3). The recombinant strain was named S288C-H2.

### 3.4. MVA Production and Transcriptomic Changes in Recombinant S288C-H2

S288C-H2 was grown in the same supplemented media as S288C, except for the minimum medium M1, considered not necessary in further analysis.

**Figure 4:**
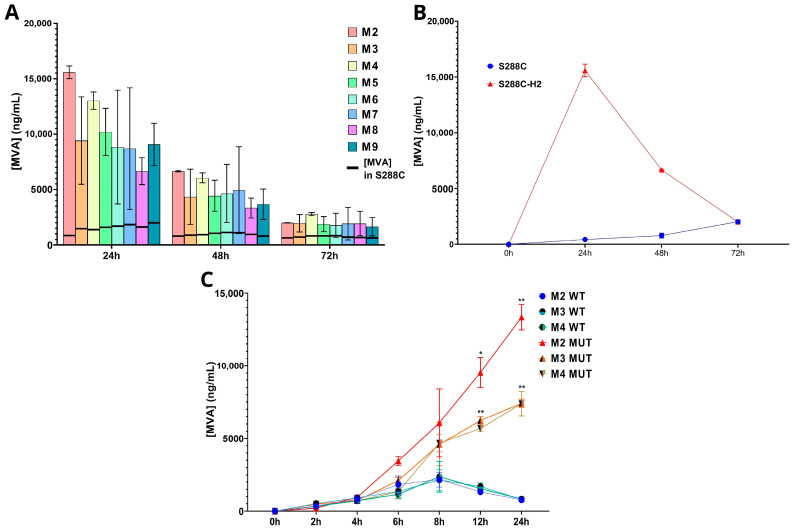
Recombinant strain S288C-H2. (**A**) MVA quantification in the 9 different media during 24 h, 48 h and 72 h. (**B**) Comparison between MVA concentration of S288C and S288C-H2 at 24 h, 48 h and 72 h. (**C**) MVA concentration in S288C-H2 (MUT) compared to S288C (WT) during the first 24 h of growth in M2, M3 and M4. * *p*-value < 0.05; ** *p*-value < 0.005.

As expected, S288C-H2 produced a higher quantity of MVA than the wild-type S288C (Figure 4A). However, in contrast with the unmodified strain, the recombinant strain seems to achieve its higher concentration of MVA in the M2 instead of M9. Because sterols exert feedback inhibition in HMG-CoA reductase, a higher production of ergosterol will lead to lower levels of MVA [51]. We speculated that the highest concentration of MVA found in the M2 medium was due to the lack of iron supplementation, which is a cofactor of many enzymes involved further in the isoprenoid biosynthetic pathway to produce ergosterol using the pool of precursors derived from MVA [24]. Thus, in the M2 medium, the activity of HMG-Coa reductase is not inhibited by the high concentration of ergosterol. After 24 h, the MVA concentration starts decreasing immensely until 72 h, where MVA values were at similar levels to the wild-type strain (Figure 4B). Although at the late stationary phase the cells maintain a stabilized production of sterol intermediates, in our S288C-H2 recombinant model, the levels of MVA drastically drop, showing that the cells are not actively producing MVA beyond 24 h. In addition, to observe the effects of Fe^2+^ supplementation in MVA production, we conducted a 24 h growth experiment, both for the recombinant and the wild-type strains using also media M3 and M4 (Figure 4C). MVA production in S288C-H2 compared to S288C is similar until 4 h, slightly higher at 6 h and 8 h and significantly higher after 12 h and 24 h. As expected, the MVA production was higher in the control medium M2 (13.6 fold), while Fe^2+^ supplementation reduces MVA production regardless of the Fe^2+^ concentration.

Therefore, to fully characterize our recombinant S288C-H2 strain and the differences at the transcriptomic level compared to the wild type, and considering that the main production of MVA is during the first 24 h of culture, we performed a comparative study between S288C and S288C-H2 in the control medium M2 at a maximum of 24 h, with sampling at 2 h, 6 h, 12 h and 24 h.

For the transcriptomic comparison, 390, 808, 1327 and 1660 genes were differentially expressed at 2 h, 6 h, 12 h and 24 h, respectively, when comparing the recombinant and the wild-type strains. To determine which pathways were enriched, a GSEA analysis was conducted.

**Figure 5:**
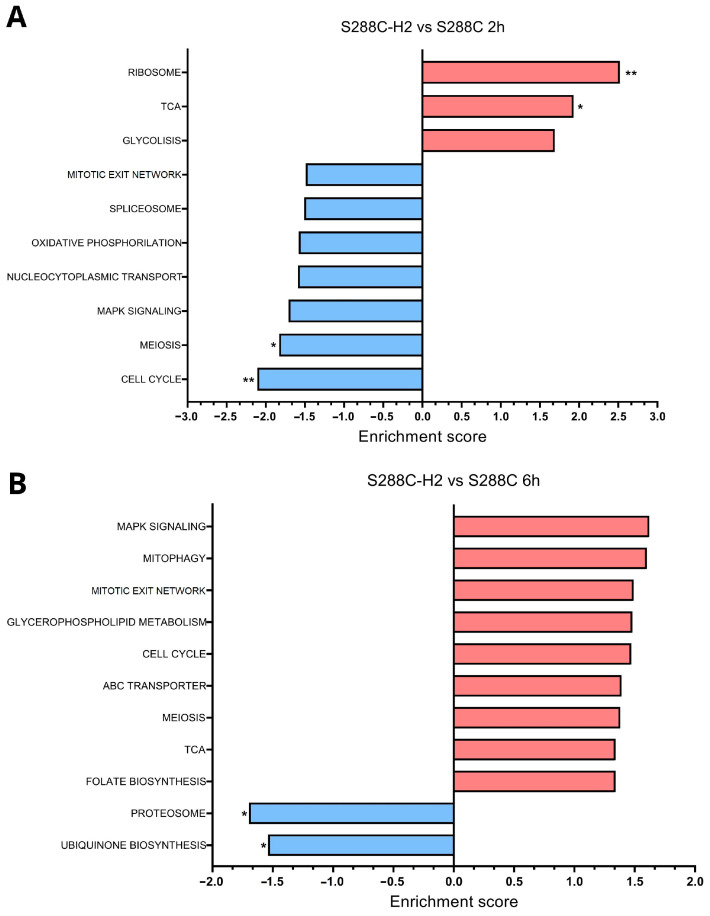
KEGG pathways enriched according the GSEA when comparing S288C-H2 against S288C grown in YPD medium at (**A**) 2 h and (**B**) 6 h. * q-value < 0.05; ** FWER q-value < 0.005.

At 2 h, we observed an upregulation in the ribosome biogenesis and the tricarboxylic acid cycle (TCA) indicating that the mutant’s need to produce more proteins and energy to support an increased biosynthetic activity. Conversely, there was downregulation in meiosis, mitogen-activated protein kinase (MAPK) signaling and cell-cycle pathways (Figure 5A). MAPK signaling and meiosis are stress-induced pathways, and their downregulation are expected as the cells were already facing glucose starvation from the previous overnight culture. The downregulation of MAPK signaling, which regulates cell-cycle progression, likely contributed to the observed cell-cycle downregulation [52,53]. Interestingly, these pathways were more downregulated in S288C-H2, potentially due to higher metabolic stress during glucose depletion. Other genes not observed in the enrichment analysis were also shown to be up or downregulated. Regarding the glycolysis pathway, an upregulation of the genes *ERR3* (phosphopyruvate hydratase), and *PDB1* and *PDA1* (subunits of pyruvate dehydrogenase) can be observed. These three proteins are involved in the conversion of glycerate-2P to acetly-CoA, the precursor of MVA. Other relevant genes that are upregulated are *FIT2* and *FIT3* (mannoproteins), involved in the iron uptake by the retention of siderophore–iron in the cell wall in an attempt to obtain iron, and *PDR5* and *PDR15* ATP-binding cassette (ABC) transporters, which are also upregulated and implicated in the detoxification of the cell, probably due to the toxic effects of steroid and terpenoid biosynthesis intermediary accumulation, such as dimethylallyl pyrophosphate (DMAPP) [54].

At 6 h, we observed a downregulation of the proteosome and ubiquinone biosynthesis (Figure 5B). The downregulation of proteosome has been previously related to the stationary phase and growth-arrested stage [55]. In our case, it can be also related to an energy-saving mechanism due to a metabolic burden, as, at this timepoint, S288C-H2 is in mid-log phase. Ubiquinone biosynthesis downregulation may be related to an excess of ubiquinone, due to its dependence on the end products of the MVA pathway, isopentenyl pyrophosphate (IPP) and DMAPP. Notably, the mevalonate kinase *ERG12*, which converts mevalonate to MVA 5-phosphate, was downregulated, possibly to avoid toxicity from accumulating MVA pathway intermediaries. This downregulation was also correlated with the downregulation of the isopentenyl-diphosphate delta-isomerase (*IDI1*) that catalyzes IPP into DMAPP. We also observed an upregulation of numerous genes related to sterol transport such as *PDR5*, *PDR15*, *RSB1* and *LAF1*, also involved in the formation of lipid droplets to store sterols and prevent lipotoxic effects from squalene, an end-product of terpenoid biosynthesis [56,57].

**Figure 6:**
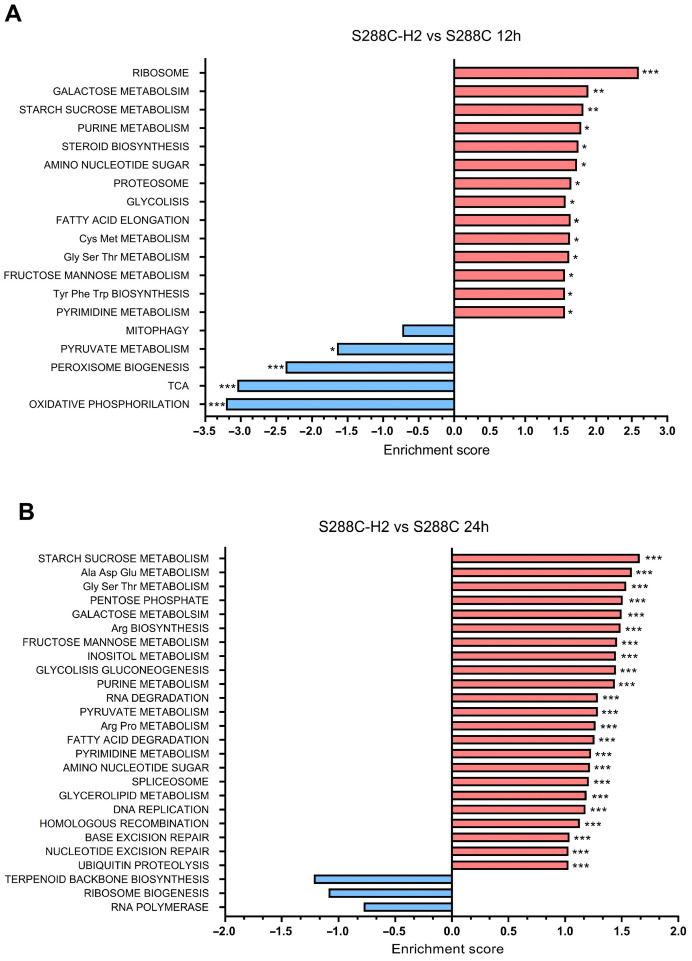
KEGG pathways enriched according the GSEA when comparing S288C-H2 against S288C grown in YPD medium at (**A**) 12 h and (**B**) 24 h. * q-value < 0.05; ** q-value < 0.005; *** q-value < 0.001.

At 12 h, eighteen pathways were affected. The upregulated pathways included ribosome biogenesis, other carbon sources’ intake, purine metabolism, steroid biosynthesis, amino sugar, nucleotide sugar, proteosome, fatty acid elongation, amino acid biosynthesis and glycolysis. The downregulated ones were oxidative phosphorylation, TCA and peroxisome biogenesis (Figure 6A). Initially, the enrichment of pathways for utilizing alternative carbon sources suggested an approaching to the diauxic shift effect. However, phosphoenolpyruvate carboxykinase (*PCK1*) and other gluconeogenesis-related genes were highly downregulated, indicating that the culture was not in the diauxic shift [58]. Moreover, the downregulation of oxidative phosphorylation, TCA and peroxisome biogenesis is directly related to an impairment on mitochondrial functions, a fact that combined with the upregulation of the mentioned pathways led us to deduce that the culture was experiencing the Crabtree effect, characterized by a decrease in respiration and an increase in the glycolysis [59]. Based on this, we can assume that the increase in the ribosomal, nucleotide and amino acid metabolism is produced by a Crabtree effect induced by the late log phase during the metabolic burden produced by the constant production of mevalonate [60]. Reduced mitochondrial activity, with lower reactive oxygen species production, also resulted in downregulated peroxisome biogenesis. Increased glycolysis would lead to the activation of other carbon source pathways. However, we did not observe increased ethanol production. Instead, we observed an upregulation in the conversion of pyruvate and ethanol towards acetaldehyde, used to produce acetate and acetyl-CoA by the genes *PDC1*, *ADH6*, *ALD3* and *ACS2*, respectively, probably caused by the overconsumption of acetyl-CoA induced by the overexpression of our mutant *HMG1*. Additionally, previous studies in yeast, cultured to achieve the Warburg effect observed in tumoral cells, have shown increased sterol and terpenoid production [61,62], which explains the upregulation in the steroid biosynthesis pathway.

Finally, the comparison at 24 h revealed a different expression of 1660 genes. GSEA revealed an upregulation of twenty-three pathways and no significant downregulated pathways. Most of these upregulated pathways were related to central carbon metabolism, amino acid metabolism and DNA repair (Figure 6B). The upregulation of the central carbon metabolism pathway as glycolysis/gluconeogenesis and galactose or fructose metabolism is a key indicator of either, Crabtree effect or diauxic shift, due to glucose depletion [41,59]. Though, in contrast to the culture at 12 h, here we observed that *PCK1* was upregulated. Moreover, we also observed an upregulation of *HSP12* (heat shock protein 12), involved in maintaining membrane organization during glucose depletion. *PCK1* and *HSP12* are involved in survival and stress response during ethanol accumulation and glucose depletion and are highly related to gluconeogenesis and diauxic shift, respectively [58,63]. A thorough examination into the genes related to the central carbon metabolism evinced an upregulation of the alcohol-dehydrogenase 2 (*ADH2*) and three aldehyde-dehydrogenases (*ALD2*, *3* and *6*). This indicates the utilization of ethanol as an alternative carbon source releasing acetate, a process commonly observed in yeasts after the diauxic shift when glucose is no longer available [64]. In the same way, the expected increment in the amino acid metabolism has been previously described in recombinant microorganisms including *S. cerevisiae* [65,66,67]. An increase in the fluxes of amino acid metabolism is provably necessary to maintain not only the native protein production but also the recombinant protein. This stress, indirectly caused by the recombinant protein or the metabolic burden, keeps draining the amino acid pool of the cell. Finally, the upregulation of DNA repair pathways is also expected as it has been documented that carbon source-starved yeast cells suffer an increase in spontaneous double-strain break formation [68].

## 4. Conclusions

The application of a combined transcriptomic and targeted metabolite analysis during CRISPR/Cas9 design and post-editing has demonstrated significant potential in both steps. In this study, pre-editing omics analyses helped in selecting top cultures for maximum mevalonate production and identifying rate limiting enzymes, revealing potential engineering targets. Post-editing analyses assisted in re-selecting culture media based on the phenotype and transcriptome changes between *Saccharomyces cerevisiae* S288C and S288C-H2, as well as in the fully characterization of the behavior of the recombinant strain.

Transcriptomic analysis post-editing provides valuable insights in gene expression differences in S288C-H2. For instance, at 6 h, the downregulation of gene *ERG12* prevents the accumulation of cytotoxic terpenoid intermediaries, leading to an exponential increase in intracellular mevalonate, confirmed by the metabolite analysis. However, the metabolic burden of constant *HMG1* expression triggers the Crabtree effect at 12 h, increasing sterol biosynthesis and consuming the produced mevalonate. In addition, the decrease in MVA production due to glucose starvation at 24 h, results in a dramatic fall of MVA concentration.

The metabolic burden produced by the overexpression of *HMG1* enhances the TCA cycle and the acetyl-CoA production. However, when the yeast enters the mid-log phase, the increased ATP demand leads to the Crabtree effect. Increasing glucose concentration in the culture medium and upregulating TCA cycle and glycolysis could maintain the yeast cells outside the Crabtree effect, further enhancing mevalonate accumulation.

Despite the promising results, challenges remain. The extensive big data volume obtained by omics approaches requires a deep analysis to understand transcriptome changes related to metabolite production. For instance, MVA is crucial for cell growth and membrane maintenance as sterol precursor. To observe changes in the ergosterol pathway, we necessitated to compare culture media with low and high productions of MVA, inducing in some of the media, a carbon starvation, thus affecting various survival pathways. Therefore, not all enriched pathways were involved in MVA production, making the selection of candidates a daunting task. Another limitation can be noticed during metabolic burden. Although this effect can be reduced, it cannot be entirely eliminated [69]. Edited cells consume more substrates, and, in the cases where the metabolite is naturally produced, it is important to consider the economic efficiency of the increased substrate consumption.

Even though our approach optimizes target region selection, there is still a long way to go. The integrative transcriptomic–metabolite analysis provides valuable insights into affected pathways and their correlations between different pathways and with the environment. However, the extensive data analysis and processing complicate the distinction of relevant pathways. Integrating artificial intelligence (AI) into metabolic engineering will present opportunities to improve data treatment [70]. AI prediction techniques used in multi-omics data can enhance the understanding of cellular metabolism, identify optimal genetic regions, resolve metabolic bottlenecks and reduce metabolic burden [69,71].

Overall, integrating transcriptomic and targeted metabolite analyses has proven its potential to optimize metabolic engineering designs. Despite the challenges in data management and pathway selection, AI integration can facilitate more efficient data processing and decision-making. We hope this approach will aid in the advancement and improvement of future metabolic engineering efforts.

## Data Availability

Raw transcriptomics data (fastq.gz files) are publicly available in ArrayExpress collection of BioStudies database (http://www.ebi.ac.uk/biostudies/arrayexpress, accessed 29 November 2024) under accession number E-MTAB-14650.

## Supplementary Materials

The following supporting information can be downloaded at: https://www.mdpi.com/article/10.3390/biom14121536/s1, Table S1 (composition of culture media), Table S2 (name and sequence of oligonucleotides used in this study), Figure S1 (original electrophoresis gel images of Figure 2B), Figure S2 (map of plasmid and gDNA sequence), and Figure S3 (relative abundance of HMG1 in wild-type and recombinant strains). Supplementary information.xlsx includes the count matrix of extracted RNA expression from wild-type S288C and mutant S288C-H2 grown in media M1 and M2 at 24 h (Supplementary Table S3) and grown in M2 at 2 h, 6 h and 12 h (Supplementary Table S4). Supporting raw data (fastq.gz files) are available in the ArrayExpress collection of BioStudies database (http://www.ebi.ac.uk/biostudies/arrayexpress, accessed 29 November 2024) under accession number E-MTAB-14650.
