# Supplementary material for: Integrative Transcriptomic and Target Metabolite Analysis as a New Tool for Designing Metabolic Engineering in Yeast
Source: Biomolecules. 2024 Nov 30;14(12):1536. doi: 10.3390/biom14121536 (PMC11673430; doi:10.3390/biom14121536)

SUPPLEMENTARY INFORMATION

Supplementary Information

Integrative Transcriptomics and Target Metabolite Analysis as a New Tool for Designing Metabolic Engineering in Yeast

Alejandro Lopez-Barbera [1], Nerea Abasolo [1], Helena Torrell [1], Nuria Canela [1] and Salvador Fernández-Arroyo [1,*]

[1] Centre for Omic Sciences, Eurecat, Centre Tecnològic de Catalunya, Joint Unit Eurecat - Universitat Rovira i Virgili, Unique Scientific and Technical Infrastructure (ICTS), 43204 Reus, Spain.

* Correspondence: Salvador Fernández Arroyo, Centre for Omics Sciences, Avda. De la Universitat, 1, 43204 Reus (Tarragona), Spain; e-mail: salvador.fernandez@eurecat.org; Phone: (+34) 977 770 959

Supplementary Table S1. Composition of culture media

MEDIUM	COMPOSITION
**M1**	YNB (0.67 g/L Yeast Nitrogen Base with amino acids)
**M2**	YPD (10 g/L Yeast extract, 20 g/L Peptone, 20 g/L Glucose)
**M3**	YPD + 54 µM $FeSO_4 \cdot 7\ H_2O$ + 10 g/L Glucose
**M4**	YPD + 54 µM $FeSO_4 \cdot 7\ H_2O$ + 10 g/L Glucose + 5 µM Calcium Pantothenate
**M5**	YPD + 54 µM $FeSO_4 \cdot 7\ H_2O$ + 10 g/L Glucose + 10 µM Calcium Pantothenate
**M6**	YPD + 54 µM $FeSO_4 \cdot 7\ H_2O$ + 10 g/L Glucose + 1 mM Sodium Pyruvate
**M7**	YPD + 54 µM $FeSO_4 \cdot 7\ H_2O$ + 10 g/L Glucose + 2 mM Sodium Pyruvate
**M8**	YPD + 54 µM $FeSO_4 \cdot 7\ H_2O$ + 10 g/L Glucose + 5 µM Calcium Pantothenate + 1 mM Sodium Pyruvate
**M9**	YPD + 54 µM $FeSO_4 \cdot 7\ H_2O$ + 10 g/L Glucose + 10 µM Calcium Pantothenate + 2 mM Sodium Pyruvate

Supplementary Table S2. Name and sequence of selected oligonucleotides, indicating their use.

NAME		SEQUENCE	USE
**UpTEF1**	F'	CCCACACACCATAGCTTCAA	Cloning
	R'	GCCTTTTTCGACGAAGAAAAAGA	
**DownADH1**	F'	GATATCCTTTTGTTGTTTCCGGGT	Cloning
	R'	TGTATATGAGATAGTTGATTGTATGCT	
**HMG1**	F'	ATGCCGCCGCTATTCAAGG	Cloning
	R'	TTAGGATTTAATGCAGGTGACGGA	
**OE-TEF1**	F'	CCCACACACCATAGCTTCAAAATGTTTCTACTCC	Overlap Extension
	R'	GGAAACAACAAAAGGATATCGCCTTTTTCGACGAAGAAAAAGAAACG	
**OE-ADH1**	F'	CGTTTCTTTTTCTTCGTCGAAAAAGGCGATATCCTTTTGTTGTTTCC	Overlap Extension
	R'	GTCCCTTGAATAGCGGCGGCATTGTATATGAGATAGTTGATTG	
**OE-HMG1**	F'	CAATCAACTATCTCATATACAATGCCGCCGCTATTCAAGGGAC	Overlap Extension
	R'	TTAGGATTTAATGCAGGTGACGGACCCATCTTTCAAACGA	
**HR-TAHMG**	F'	CGACAAAGATTTTGTTATCGGCTTTATTGCTCAAAGAGACATTAATGAACTCCCACACACCATAGCTTC	URA3 Homology regions
	R'	ATCCACGGTTCTATACTGTTGACCCAATGCGTCTCCCTTGTCATCTAATTAGGATTTAATGCAGGTGA	
**gDNA_URA3**	F'	GATCATTGATTATGACACCCGGTGTGTTTTAGAGCTAG	Guide DNA inserted in Pml104-KanMx
	R'	CTAGCTCTAAAACACACCGGGTGTCATAATCAA	
**URA3**	F'	CCTAGTCCTGTTGCTGCCAA	URA3 region vertification
	R'	TGTCATCTAAACCCACACCGG	
**pML104**	F'	GCAATTAACCCTCACTAAAGG	Sanger pML104

Supplementary Figure S1. Original electrophoresis gels to obtain Figure 2B in the main manuscript. Lanes inside a red box in the panel B were added to the panel A according to the ladder height to obtain the final figure.

A

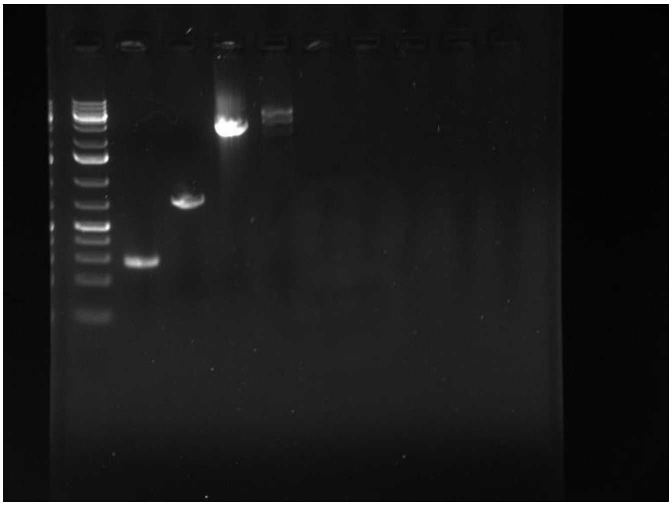

B

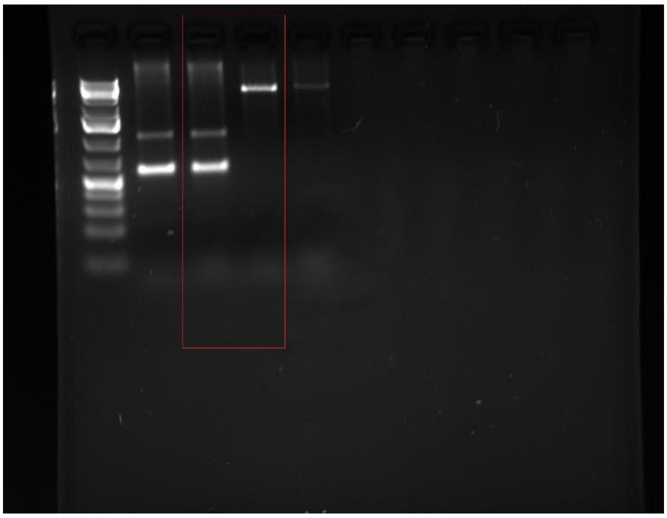

Supplementary Figure S2. Map of pML104-KanMx4 plasmid, indicating the insertion of the gDNA sequence. The map was generated using the online tool PlasMapper 3.0 (https://plasmapper.wishartlab.com/, accessed 29 November 2024), indicating the sequence of the pML104-KanMx4 plasmid obtained from Addgene (http://addgene.org/83476, accessed on 29 November 2024).

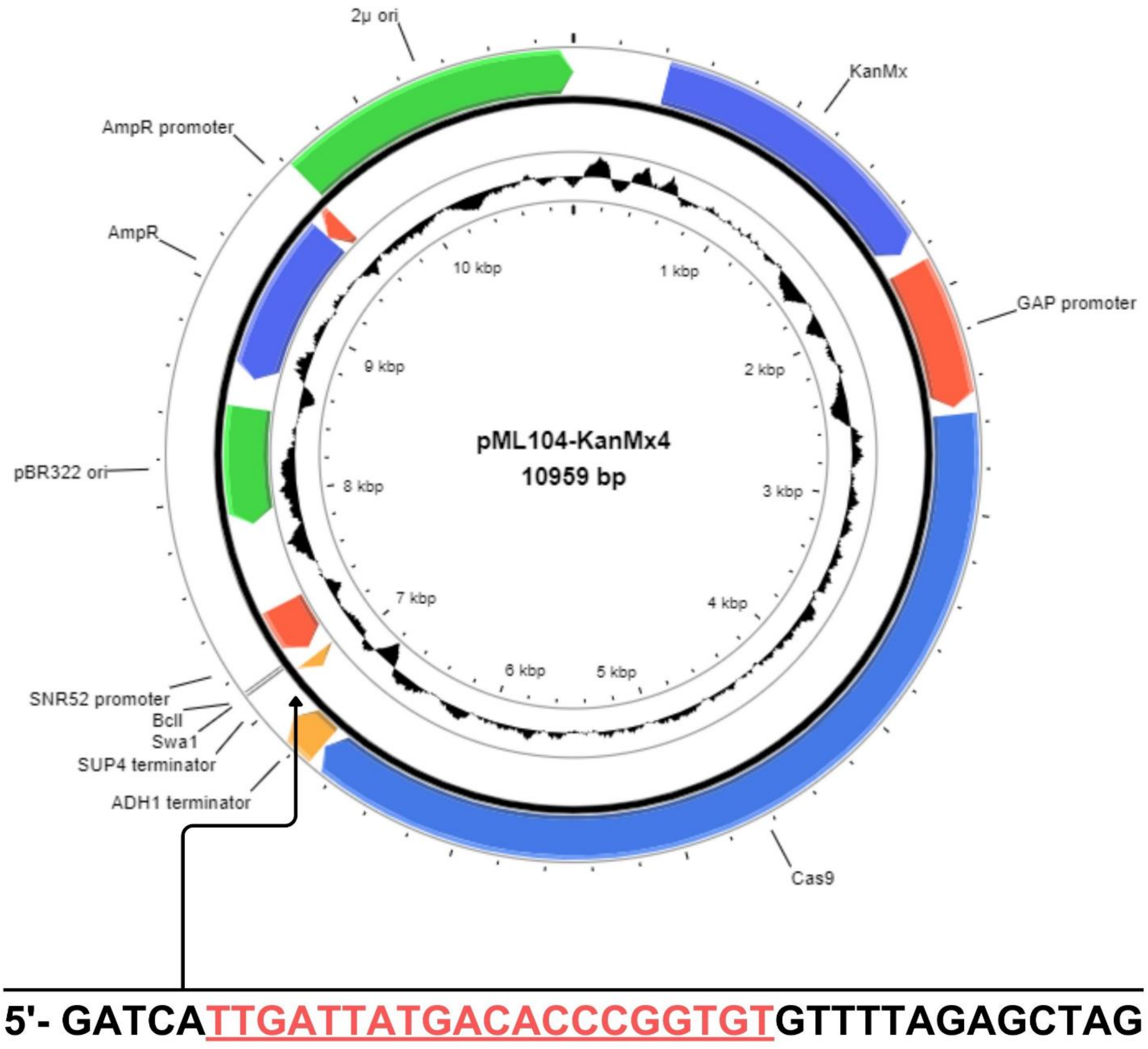

Supplementary Figure S3. Differences in gene expression of HMG1 in S288C and S288C-H2 after data normalization using the reference gene ACT1 (p-value < 0.03).

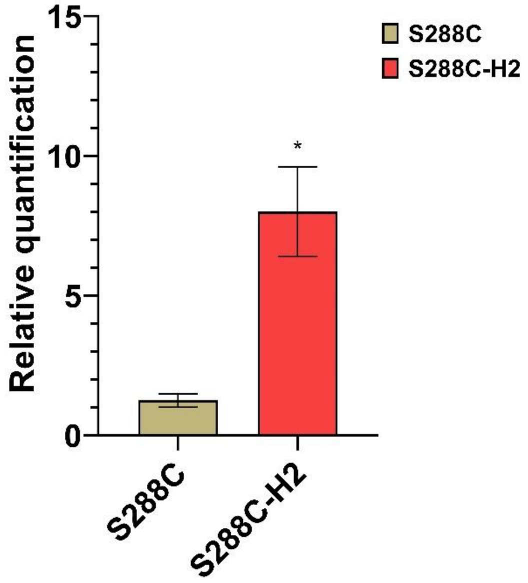

Supplement: Supplementary file 1 [file biomolecules-14-01536-s001.zip › Supplementary Information.pdf]
